# Individual Zn_2_SnO_4_-sheathed ZnO heterostructure nanowires for efficient resistive switching memory controlled by interface states

**DOI:** 10.1038/srep03249

**Published:** 2013-11-19

**Authors:** Baochang Cheng, Zhiyong Ouyang, Chuan Chen, Yanhe Xiao, Shuijin Lei

**Affiliations:** 1 School of Materials Science and Engineering, Nanchang University, Jiangxi 330031, P. R. China; 2 Nanoscale Science and Technology Laboratory, Institute for Advanced Study, Nanchang University, Jiangxi 330031, P. R. China

## Abstract

Resistive switching (RS) devices are widely believed as a promising candidate for next generation nonvolatile resistance random access memory. Here, Zn_2_SnO_4_-sheathed ZnO core/shell heterostructure nanowires were constructed through a polymeric sol–gel approach followed by post-annealing. The back-to-back bipolar RS properties were observed in the Ohmic contact two-terminal devices based on individual core/shell nanowires. With increasing bias to about 1.5 V, it changes from high-resistance states (HRS) to low-resistance states, and however, it can be restored to HRS by reverse bias. We propose a new mechanism, which is attributed to the injection of electrons into/from interfacial states, arising from the lattice mismatch at ZnO/Zn_2_SnO_4_ heterointerface. Upon applying negative/positive voltage at one end of devices, where interfacial states are filled/emptied, barrier will be eliminated/created, resulting into symmetric RS characteristics. The behavior of storage and removal charges demonstrates that the heterostructures have excellent properties for the application in resistance random access memory.

Resistive switching (RS) behavior has attracted increasing attention due to its application in the next generation nonvolatile resistance random access memory (ReRAM). The RS behavior originates from repeated resistance changes between high resistance state (HRS) and low resistance state (LRS), and is controlled by an external electrical field with opposite polarity. This type of memory devices, generally composed of metal-insulator-metal structures, has many merits for applications such as high-density, high-speed, and low-power consumption. Till now, the RS phenomena have been observed in a variety of materials such as transition metal oxide (TMO)^1^,^2^,^3^,^4^,^5^,^6^,^7^, perovskite oxide^8^,^9^,^10^,^11^,^12^, and organic polymers^13^. Moreover, some mechanisms, such as formation/rupture of conducting filament^14^,^15^,^16^, alteration of Schottky barrier^17^, migration of oxygen vacancies^18^,^19^, trapping/detrapping of charge carriers^20^,^21^, and Mott transition^22^, have been introduced to explain the origin of RS properties.

Owing to a wide direct bandgap of 3.37 eV and a large exciton binding energy of 60 meV, ZnO is an important material, ideally suited for applications in optoelectronic and electronic devices. Recent researches have shown that ZnO-based thin films have good RS characteristics as well and are very promising for ReRAM application. Bipolar RS characteristics have been observed in TiN/ZnO/Pt, Ag/ZnO/Pt, Au/ZnO/ITO, Cr/ZnO/Pt, Cu/ZnO/Pt, and Al/ZnO/Si film devices^23^,^24^,^25^,^26^,^27^,^28^. Additionally, Zn_2_SnO_4_ is also an n-type transparent conducting oxide (TCO) material with a bandgap of 3.6 eV and an electron mobility of 10–15 cm^2^ V^−1^s^−1^ 29. When Zn_2_SnO_4_ is coupled with ZnO, II-type heterostructure can be formed at their interface since its conduction band (CB) is lower than that of ZnO^30^. Thus, electron-hole pairs can be separated, and consequently, electrons will migrate to Zn_2_SnO_4_ while holes will migrate to ZnO.

For traditional RS memory devices, it is difficult to realize the scale-down of multibit storage to the size of a single memory cell due to the geometric configuration of these devices, and furthermore, the high operating voltage also limits the applications of such devices in integrated circuits. However, two-terminal one-dimensional (1D) nanostructure based RS memory devices have the great advantages of a simple fabrication process, lower power consumption, fast write/read speed and high-density storage, and therefore, numerous efforts have been devoted to the construction of high-performance RS memory devices from 1D nanostructures at present^31^,^32^. In spite of extensive studies, the underlying physical mechanism, especially on the nanoscale, is still highly controversial. For nanostructures, surface states are very important in determining their physical properties due to very large surface-to-volume ratio. In particular, Fermi level pinning at surface states is expected which would result in band bending and depleted regions in the vicinity of surfaces and/or interfaces. In order to meet the ever-increasing demand in device performance, it is necessary to tailor the properties of semiconductors which can be done by design and controlled fabrication of new nanostructures. In contrast, the application of composite oxides has been rarely explored although single TMOs have been under extensive research. In comparison with single oxides, composite oxides can form heterojunction, which have more freedom to tune the physical properties of the heterostructure by selecting the materials with different energy bands.

In this work, we demonstrate two-terminal nonvolatile RS memory devices based on Ohmic contacted Ag-ZnO/Zn_2_SnO_4_-Ag structures, where ZnO/Zn_2_SnO_4_ core/shell radial heterostructure nanowires were grown by a polymeric sol–gel approach followed by a post-annealing. The DC voltage bias was applied to the two ends of Ag electrodes during the electrical measurements. At ZnO/Zn_2_SnO_4_ heterointerface, the lattice mismatch results into the presence of interface states, served as a charge trap or mediator, namely, the storage medium for the memory.

## Results

**Figure 1:**
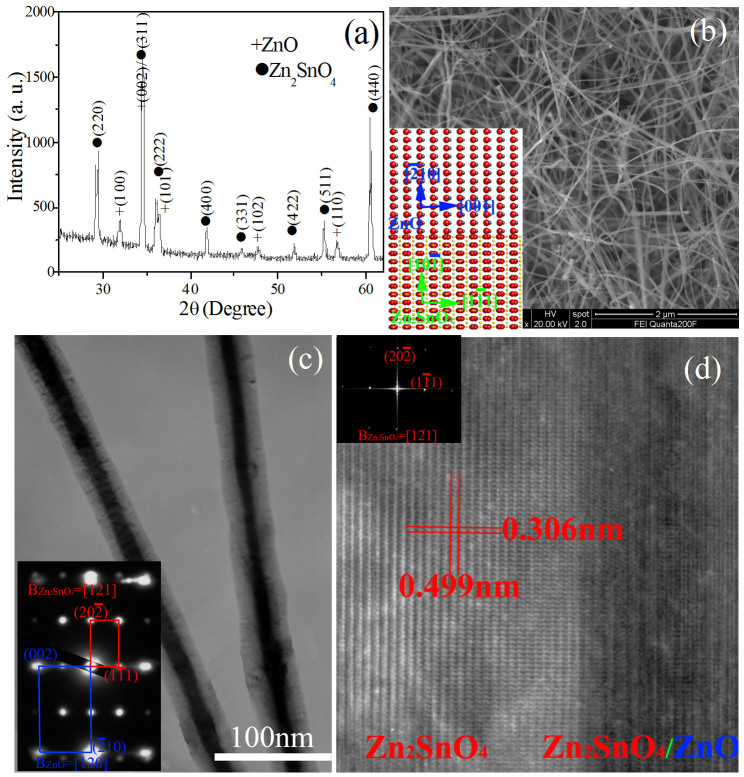
Structural characterization of ZnO/Zn_2_SnO_4_ core/shell heterostructure nanowires. (a) XRD pattern of the product, showing that it is composed of Zn_2_SnO_4_ and ZnO. (b) FESEM image. (c) Low-magnification bright-field TEM micrograph of ZnO/Zn_2_SnO_4_ nanowires, showing a typical core/shell heterostructure with dark core and light shell. (d) High-resolution TEM image, taken from the core/shell heterostructure interface area, the inset in (d) corresponds to the FFT pattern of outer shell.

The X-ray diffraction (XRD) pattern [Fig. 1(a)] of the as-grown product presents clear evidence that the nanostructures are composed of two crystalline phases, that is, diamond-cubic Zn_2_SnO_4_ (JCPDS file: 24-1470) and wurtzite-hexagonal ZnO (JCPDS file: 36-1451). No characteristic peaks from other crystalline forms are detected in the XRD pattern. Low-magnification field-emission scanning electron microscopy (FESEM) observation (Figure 1b) reveals that the as-prepared product consists of a great quantity of wire-like nanostructures with typical lengths in the range of several to several tens of micrometers. These wire-like structures are randomly oriented, and most of them are slightly bent. Shown in Fig. 1(c) is the typical low-magnification bright-field transmission electron microscopy (TEM) image of the as-grown product. The product displays different contrasts from edge to core. The nanostructures are sheathed with the outer layer of a shallow contrast along the “wire” axis directions and the middle layer shows darker contrast, suggesting the formation of core/shell heterostuctures. The sharp interface between inner core and outer wall clearly shows that ZnO nanowires are fully sheathed by Zn_2_SnO_4_ layer along the entire length. The selected area electron diffraction (SAED) pattern [inset in Fig. 1(c)] indicates a superposition of two sets of single-crystal diffraction spots, which can be indexed to cubic Zn_2_SnO_4_ with [121] zone axis and hexagon ZnO with [120] zone axis, revealing the epitaxial relationship of 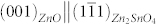, 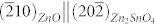 and 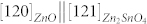. The corresponding structure model of hexagon ZnO (upper) and cubic Zn_2_SnO_4_ (lower) projected along [120]*_hexagon_* and [121]*_cubic_* is inset in Figure 1b and clearly reveals their orientation relation. ZnO has a hexagonal crystal structure with lattice constants a = 0.3249 and c = 0.5206 nm, and Zn_2_SnO_4_ has a cubic crystal structure with lattice constants a = 0.8650nm. The lattice mismatch is about −10% between (001)*_ZnO_* and 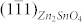 and −6.2% between 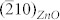 and 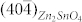 (in reference to ZnO). Therefore, there exist quantities of stress at the ZnO/Zn_2_SnO_4_ interface owing to the lattice mismatch. Figure 1d is a high-resolution TEM image, taken from the heterostructure interface region of an individual ZnO/Zn_2_SnO_4_ nanowire, which shows the detailed interface structure between the inner ZnO core and outer Zn_2_SnO_4_ shell. The fast Fourier transform (FFT) analysis [inset in Fig. 1(d)], taken from the outer shell, can be indexed to a cubic Zn_2_SnO_4_ with [121] zone axis. Due to the coating of Zn_2_SnO_4_ on the surface of ZnO nanowires, the atom resolution image of inner core cannot be observed effectively. Due to the incorporation of some Sn atoms into ZnO lattice, additionally, the growth of ZnO is along 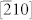 direction rather than c-axis [001].

**Figure 2:**
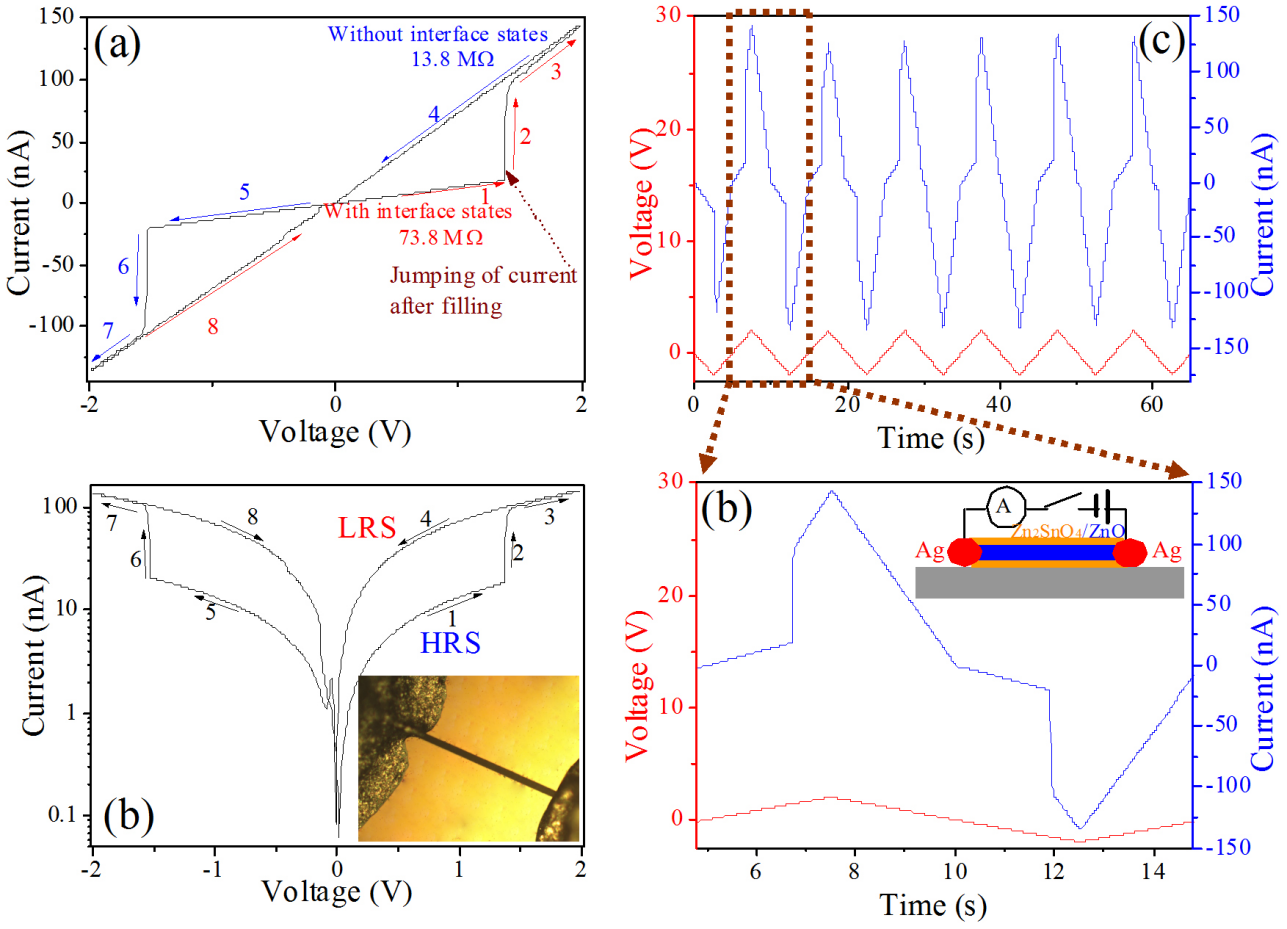
(a) Typical I-V characteristics of the Ag-ZnO/Zn_2_SnO_4_-Ag RS memory device in linear scale, the numbered arrows (1–8) indicate the sweep direction. (b) I-V characteristics in semilogarithmic scale, the inset in (b) shows an enlarged optical image of the RS memory device based on an individual core/shell heterostructure. (c) Under voltage sweeps, the current response (blue curve) to voltage (red curve), and applying triangle wave voltage with an amplitude of 2.0 V and a frequency of 0.1 Hz. (d) An enlargement of a brown dashed frame in (c), the inset shows the schematic structure for the electrical measurement of the device.

The enlarged optical image and the schematic structure of the RS memory are respectively inset in Fig. 2(b) and (d), which consist of a ZnO/Zn_2_SnO_4_ nanowire that is in contact with Ag electrodes on an Al_2_O_3_ substrate. The typical two-terminal current-voltage (I-V) characteristics of the Ag-ZnO/Zn_2_SnO_4_-Ag memory cell are described both in linear and semilogarithmic scales, as shown in Fig. 2(a) and (b). The electrical measurement was performed by a DC voltage sweep mode at a frequency of 0.1 Hz at room temperature and all the bias voltages were applied to the two end Ag electrodes of heterostructure nanowires. As can be seen, the voltage was swept in a range of −2 to 2 V. It is observed that, with the increase of the applied voltage from 0 to 1.5 V (part 1), the device is originally in the high-resistance state (HRS) and then the output current of the device increased abruptly at about 1.5 V, switching from HRS to low-resistance state (LRS). Thus, a set process occurs at about 1.5 V (V_set_). Afterward, when the voltage was swept to a negative value, the device current is still LRS (part 4). As the voltage is subsequently decreased toward negative values from 0 to −1.5 V (part 5), however, the device current shows a high-resistance OFF state, indicating that the formed low-resistance ON state can be eliminated by being applying a reverse bias. When the reverse bias voltage exceeded a certain negative value of about −1.5 V, the device changes back to the low-resistance ON state. Therefore, the resistance change is almost symmetric when the voltage sweeps cyclically. At about 1.5 V, we obtain an extremely large memory window (R_HRS_/R_LRS_) which makes the device easy to be utilized. The resistance switching ratio is given by 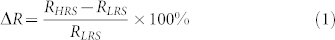
where R_HRS_ and R_LRS_ denote the high resistance state and low resistance state, respectively. According to the Equation 1, the resistance switching ratio of the ZnO/Zn_2_SnO_4_ nanowire is about 4.4.

**Figure 3:**
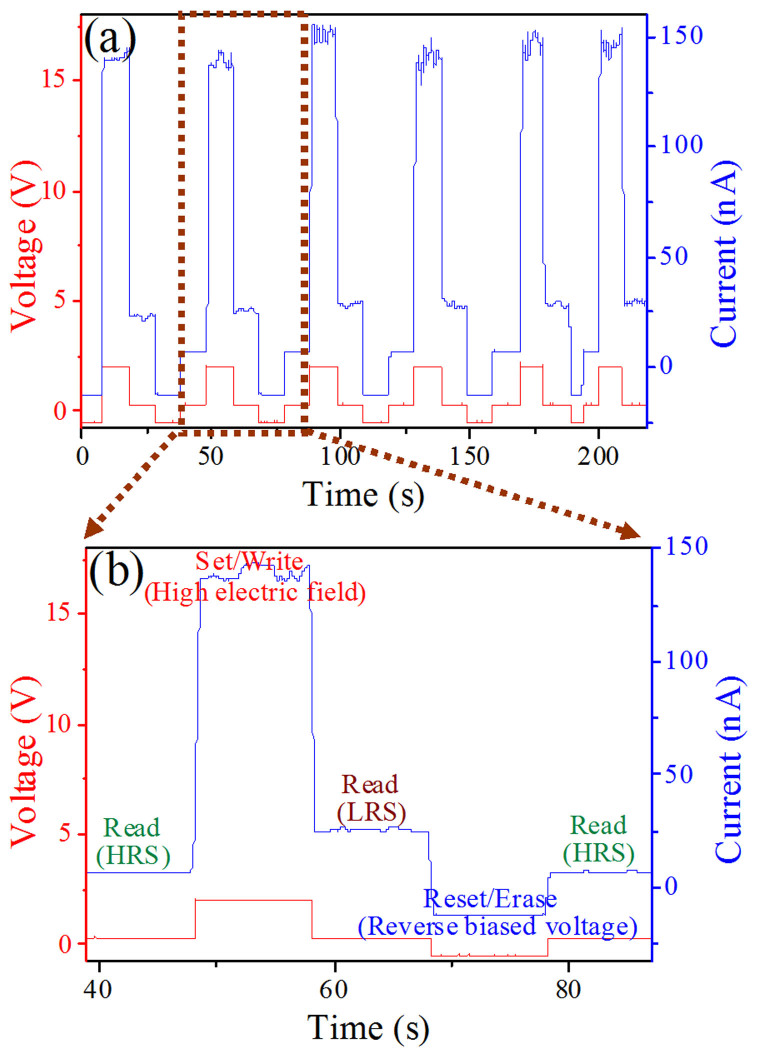
(a) Write/read access of RS cell as a memory, the red curve corresponding to applied voltage and the blue curve corresponding to respondent current. (b) an enlargement of a brown dashed frame in (a), showing a detail set/reset access, the information can be written/set by a relatively high voltage of 2 V, read by a relatively low voltage of 0.3 V, and erased/reset by a reverse biased voltage of −0.5 V.

The two-terminal Ag-ZnO/Zn_2_SnO_4_-Ag Ohmic contacted devices exhibit a symmetric resistance switching characteristic upon being applied a cyclic sweep voltage. More interestingly, however, the forward resistance of device can be restored to pristine HRS after being applied a reverse bias voltage, namely, reset process. Therefore, they can be used as RS memory, and the measurement is shown in Fig. 3. It can be seen that the information can be set/written by applying a relatively high voltage of 2 V at the two ends of Ag electrodes, read by applying a relatively low voltage of 0.3 V, and reset/erased by applying a reverse bias voltage of −0.5 V.

Resistive memory requires high reliability for practical applications. To verify the reproducibility and stability of the RS effects, the repeating sweep cycling was performed on the Ag-ZnO/Zn_2_SnO_4_-Ag memory cell. As shown in Fig. 3, a single selected cell was tested under the DC sweeping mode by conducting a series of successive set/reset cycles. It is observed for 8 repeated switching.

## Discussion

**Figure 4:**
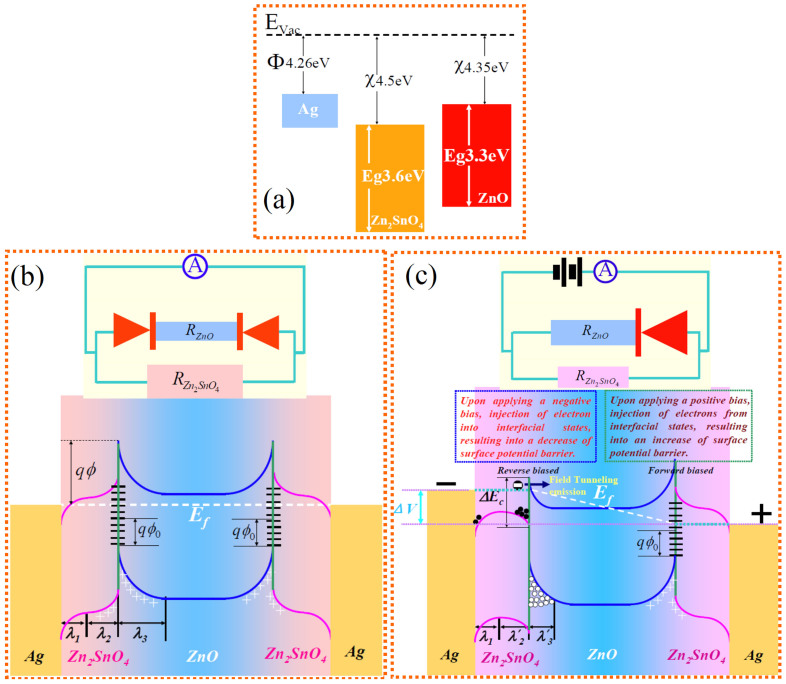
Schematic representation of the band alignment for RS of n-type Zn_2_SnO_4_/n-type ZnO core/shell heterojunction. (a) Before contact. (b) At a zero or relatively low bias voltage, the presence of interface states induces the depletion region with high barrier near the heterointerface, and forms a back-to-back contacted diode for the core ZnO. (c) At a relatively high bias voltage, the interface states are filled near the electrode subjected to a negative voltage, resulting into a disappearance of interface potential barrier, the device changes from HRS into LRS. However, the interface states are created near the electrode subjected to a positive voltage, and correspondingly, electrons are injected from the interface states into the electrode, and the interface potential barrier increases and the energy band moves upward. The insets on the top of (b) and (c) are the schematic diagrams of corresponding electric circuit.

From the resistance change, the RS behavior of the memory cell seems to exhibit a quasi-unipolar RS nature since the device can both be switched to the LRS in the two different directions of nanowire-based memory cell. Compared with the classic unipolar RS, however, the reset process does not occur in the same bias direction as the set process. In additional, LRS in a forward direction can be restored to HRS by being applied a reverse voltage. These results indicate that the device is composed of two back-to-back bipolar RSes rather than a single classic unipolar or bipolar RS, and furthermore, they exist near the two end electrodes of device, respectively. Although the nature of RS and related charge transport process on microscale in meta-semiconductor-metal structures has been extensively proposed^14^,^15^,^16^,^17^,^18^,^19^,^20^,^21^,^22^, the core/shell NW-based RS is obviously different from the classic unipolar and bipolar RS. The electronic injection into/from interface states, controlled by being applied a bias voltage, should be a dominant mechanism in our RS nanodevices. In light of the above structural characterization, the principle of the Ag-ZnO/Zn_2_SnO_4_-Ag memory cell can be easily understood from the energy-band diagram depicted schematically in Fig. 4. The electron affinity of ZnO (*χ_ZnO_* = 4.35 *eV*) is lower than that of Zn_2_SnO_4_ (*χ_ZnO_* = 4.5 *eV*)^33^. When they contact each other, therefore, II-type band alignment would be obtained based on their electron affinity and energy gap calculation. However, they are both n-type wide bandgap semiconductors because of the oxygen vacancies in an un-doped ZnO and Zn_2_SnO_4_^34^, and moreover, the lattice mismatches at their heterointerface, verified by the TEM and SAED analysis, Thus, quantities of acceptor-type centers exist at their heterointerface, and thus the electrons in nanowire interior can diffuse onto the interface states, and correspondingly, the CB and valence band (VB) bend upward and simultaneously form a low-conductivity depletion layer near their interface, that is, the formation of interface states. As a consequence, the interface potential barrier prevents charges from freely passing through at their heterointerface in the equilibrium state^35^. Because the work function of Ag (Φ*_Ag_* = 4.26 *eV*) is lower than the electron affinity of n-type Zn_2_SnO_4_^36^, it can form Ohmic contact when Ag is in contact with ZnO/Zn_2_SnO_4_ heterostructures, and moreover, the I-V curves both exhibit linear characteristics under OFF and ON states. Therefore, there exists no barrier at the Ag electrodes and heterostructure nanowire interface, and the Zn_2_SnO_4_ shell only serves as a resistor.

When one end of the device is applied a negative bias voltage, the direction of external electrical field is just the same as the electron diffusion direction of nanowire interior, namely reverse bias. Therefore, it is difficult for electrons to cross the heterointerface at a relatively low negative bias voltage. As a consequence, only the Zn_2_SnO_4_ shell can contribute to the conductivity, and the device shows a HRS. At a relatively high bias voltage, however, the external electrons can be injected into interface states from the electrode applied a negative voltage and then captured by acceptor-type centers, resulting into the filling of interface states by the external electrons, and correspondingly, the electronic drift increases toward the interior of nanowires, and the energy band moves downward. As a result, the interface potential barrier height and the depletion layer width decrease. The effective barrier height *ϕ_eff_* is related to the depletion width (*λ*) on the two sides of the interface as^19^,^20^,^21^

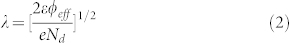
where *e* is the electronic charge, *N_d_* is the doping density, and *ε* is the dielectric constant of semiconductor.

When the depletion width *λ* is lower than the mean free path of electrons, it will be very easy for electrons to cross the interface by tunneling mechanism^35^. With further increasing negative bias voltage, the interface potential barrier will eliminate eventually. For the core ZnO, it is highly doped by Sn. At a relatively low bias voltage, its nonequilibrium carrier density is much lower than the doping concentration. Once the surface potential barrier eliminates, almost all of the external electrical field will be imposed on the core ZnO, which can induce impurities to ionize, resulting into a rise of Fermi level. Moreover, it is very easy for Fermi level to exceed CB level, leading to a formation of degenerate semiconductor. At this transition point, therefore, *R_ZnO_* decreases as well. This process of resistance mutation leads to the ON state (LRS) for the memory, corresponding to a write/set operation for the memory. In ON state, the Zn_2_SnO_4_ shell and ZnO core both contribute to the conductivity, and the overall macroscopic resistance of memory cell can be expressed as 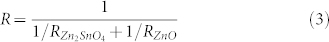
where 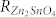 and *R_ZnO_* correspond to the resistance of Zn_2_SnO_4_ and ZnO, respectively. Because their Fermi levels of n-type ZnO core and Zn_2_SnO_4_ shell are both higher than their CB levels, namely, degenerate semiconductor. Therefore, their currents can both show linear properties before and after switching.

On the contrary, when the other end of the device is subjected to a positive voltage, electrons will be injected from the interface states into the electrode, and thus, the electrons in nanowire interior will diffuse into the acceptor centers of interface states. Correspondingly, the interface potential barrier height and depletion layer width increase. Although the interface potential barrier rises, it is forwardly biased, and hence, electrons can pass through this interface barrier if the applied voltage direction remains unchanged. However, when this electrode is re-subjected to a relatively low negative voltage, this barrier will be reversely biased, and consequently, the device can be switched back to the high-resistance OFF state. This indicates that the written information in one direction can be erased by being applied a reverse voltage. With the magnitude of the negative bias exceeding to the threshold filling, the interface states will be filled again and then the device is changed back to the ON state, indicating that the information in core/shell nanowire-based memory cell can both be written in two different directions by be applying a relatively high voltage. It is due to the injection of electrons into/from interface states, controlled by being applied negative/positive bias that the interface states can be eliminated/created. As a consequence, the device exhibits ON/OFF state, which enable core/shell nanowires to be used as memory and store information. Furthermore, Zn_2_SnO_4_ shell and ZnO core both contribute to the device conductivity in the ON state, while only Zn_2_SnO_4_ shell contributes the device conductivity in the OFF state. At a relatively high DC voltage, additionally, the interface states will be filled near the electrode subjected to a negative voltage, while it will be created near the one subjected to a positive voltage, and therefore, the resistance of the two-terminal device can show a symmetric variation upon being applied a cyclic sweep voltage.

In summary, we have demonstrated that the nanowire-like core/shell radial heterostructures can not only show RS characteristics but serve as a memory in two-terminal Ohmic contact devices. The devices show high performance of their storage characteristics such as their high-resistance on/off ratio and low operating voltage. It is different from the classic unipolar and bipolar RS, which is composed of two back-to-back bipolar RSes. The device resistance changes symmetrically when the applied voltage sweeps cyclically. With increasing voltage to about 1.5 V, its resistance changes from HRS into LRS, and then the resulting LRS can change back to HRS by being applied a reverse voltage. The interface states, resulting from the lattice mismatch at ZnO/Zn_2_SnO_4_ heterointerface, dominate the conduction mechanisms of RS. The injection of external electrons into/from the acceptor-type interface states when the end electrode of heterostructure nanowires is subjected to a relatively high negative/positive voltage, resulting into the elimination/creation of interface potential barrier, which make the device function like a floating gate memory, This study demonstrated that the core/shell radial heterostructure nanowires have excellent properties for the application in the next-generation nonvolatile resistance random access memory with high-density and high-performance.

## Methods

Zn_2_SnO_4_-sheathed ZnO core/shell heterostructure nanowires employed in this work were synthesized by a polymeric sol–gel approach followed by a post-annealing. Zn(NO_3_)_2_·6H_2_O, SnCl_2_**·**2H_2_O, citric acid, and ethylene glycol were added to de-ionized water. A sol was formed at 80°C, and subsequently polymerized to a gel at 150°C. The solid gel was prepyrolyzed at 400°C to form a Zn–Sn–C–O amorphous composite precursor. The resulting precursor was ground, placed in a ceramic crucible covered with a ceramic lid, and then put into a box furnace and heated at 1000°C for 120 min. The resulting sample was studied and analyzed by X-ray diffraction (XRD, RIGAKU D/max-3b), field-emission scanning electron microscopy (FESEM, FEI Quanta 200F), and high-resolution transmission electron microscopy (HRTEM, JEOL 2010) to evaluate the crystal structure and morphology.

For the device preparation, the ZnO/Zn_2_SnO_4_ heterostructure nanowires were dispersed on an Al_2_O_3_ substrate and the two-terminal Ag contacts of an individual nanowire device are fabricated by semi-dried silver paste. And then, a post-annealing under N_2_ atmosphere at 400°C for 10 min is required to minimize the contact resistance. Electrical measurements were carried out by a synthesized function generator (Stanford Research System Model DS345) and a low-noise current preamplifier (Stanford Research System Model SR570).

## Author Contributions

B.C.C. designed and performed experiments. B.C.C., Z.Y.O., C.C., Y.H.X. and S.J.L. wrote the manuscript and B.C.C. prepared figures 1–4. All authors reviewed the manuscript.
